# A comparison of efficacy and safety of complementary and alternative therapies for severe mycoplasma pneumonia in children: A protocol for systematic review and meta-analysis

**DOI:** 10.1097/MD.0000000000023959

**Published:** 2021-02-19

**Authors:** Xiao Wang, Hongan He, Jialin Zheng, Jinjuan Wang, Hao Zheng, Baoqing Zhang

**Affiliations:** a The First College of Clinical Medicine, Shandong University of Traditional Chinese Medicine; b The Affiliated Hospital of Shandong University of Traditional Chinese Medicine, Jinan, Shandong Province, China.

**Keywords:** complementary and alternative therapy, net meta-analysis, protocol, severe *Mycoplasma pneumoniae* pneumonia in children, systematic review

## Abstract

**Background:**

In recent years, the incidence rate of children with severe *Mycoplasma pneumoniae* pneumonia (SMPP) is increasing, which poses a great threat to children's life and safety. There are some limitations in the existing drugs for the treatment of SMPP, and the supplementary and alternative therapy of SMPP plays an irreplaceable role in the treatment of this disease. This study will evaluate the efficacy and safety of various complementary and alternative therapies for SMPP by means of mesh meta-analysis. In order to provide the basis for clinical rational use.

**Methods:**

Two researchers will independently and comprehensively searched the Cochrane Central controlled trials registry, Cochrane Library, PubMed, web of science, EMBASE, CNKI, and Wanfang database to collect randomized controlled trials (RCT) studies on complementary and alternative therapies for SMPP. And the relevant references included in the systematic review/meta-analysis are screened. The retrieval time limit is from the establishment of the database to November 2020. We will use Revman 5.3 software for meta-analysis and use grade to grade the quality of evidence in the net meta-analysis (NMA).

**Results:**

The aim of this study was to compare the efficacy and safety of different complementary and alternative therapies in the treatment of SMPP, with a view to evaluating and ranking different interventions.

**Conclusion:**

The supplement and replacement therapy of SMPP can improve the clinical efficacy, relieve the clinical symptoms, improve the quality of life of children, and reduce adverse reactions, which can provide strong support for the rational use of clinicians.

**INPLASY registration number:**

INPLASY2020110079.

## 1 Introduction

Pneumonia is the main cause of death in children under 5 years old. According to statistics, nearly 1 million people die of the disease every year.^[1,2]^ Among them, *Mycoplasma pneumoniae* pneumonia (MPP) is a common acute lower respiratory tract infection disease in children, accounting for about 1/3 of the hospitalized children with community-acquired pneumonia (CAP).^[3,4]^ In recent years, the proportion has been increasing.^[5–7]^ The majority of children with MPP have mild symptoms, and the effect of macrolide antibiotics is better. However, with the emergence of *Mycoplasma pneumoniae* (MP) infections resistant to macrolides, the number of children with severe *Mycoplasma pneumoniae* pneumonia (SMPP) keeps increasing, which is more likely to complicate severe pulmonary and extrapulmonary complications.^[8,9]^ In addition to persistent or intermittent fever, cough and expectoration, clinical manifestations include obstructive bronchitis, pulmonary fibrosis, pleural effusion, acute respiratory distress syndrome, and severe symptoms of liver, kidney, brain, cardiovascular and nervous system, which pose a great threat to children's life safety.^[10]^

At present, SMPP mostly adopts combined treatment measures. Macrolide antibiotics are still the most effective and commonly used drugs against mycoplasma infection. In addition, immune preparations and bronchial lavage are effective in the treatment of this disease. *M pneumoniae* parasitizes outside the cell and does not have a cell wall. The main purpose of the application of macrolide antibiotics is to inhibit and interfere with protein synthesis. As a first-generation macrolide drug, erythromycin can reduce the production of respiratory secretions and indirectly reduce airway hyperresponsiveness due to its good antibacterial properties. It was first used for the treatment of MPP in children, but the drug can cause more obvious adverse reactions in the digestive tract, even cause phlebitis and local pain, long-term medication can cause liver and kidney damage.^[11]^ Azithromycin, as the second-generation macrolide drug, has stronger tissue penetration, longer half-life than erythromycin, and relatively fewer gastrointestinal adverse reactions. It is currently the most commonly used drug for the treatment of MPP in children.^[12]^ However, with the emergence of *M pneumoniae* resistant strains, the number of cases of *M pneumoniae* with macrolides resistant drugs increased gradually, and the overall fever days, course and hospitalization time of the children are prolonged. At the same time, there are more inflammatory reactions and complications.^[13,14]^ In addition, high-quality, large sample, and multicenter studies are needed for the treatment course, therapeutic effect, and safety of macrolide antibiotics in the treatment of mycoplasma pneumonia.^[15]^ The occurrence of SMPP is closely related to the immune mechanism. Glucocorticoid has anti allergic, anti-inflammatory, immunomodulatory, and other pharmacological effects, so the effect of glucocorticoid in the early stage of SMPP is obvious.^[16]^ However, the timing of combined glucocorticoid therapy is uncertain and needs further evaluation. Gamma globulin contains multi titer immunoglobulin G antibody, which has the dual therapeutic effects of immune replacement and immune regulation. However, the drug is expensive and may cause anaphylactic reaction, so it can be recommended for patients with obvious extrapulmonary injury.^[17]^ Fiberoptic bronchoscopy can directly observe the pathological changes of children's bronchial mucosa, and clear the pathogenic bacteria through bronchoalveolar lavage fluid. At the same time, local administration can effectively remove respiratory tract secretions and pathogens, which is often used in the auxiliary diagnosis and treatment of SMPP.^[18]^ And it has clear indications and contraindications, and by this stage, the child's condition is often more serious. Therefore, it is necessary to improve the symptoms of the children through treatment other than drugs.

Magnetic therapy, acupoint application, cupping, acupuncture, massage, traditional Chinese medicine, etc have significant effects in reducing airway sensitivity, improving blood and lymph circulation, improving clinical efficacy, relieving clinical symptoms, improving the quality of life of children, reducing adverse reactions, and so on. They play an important role in the adjuvant treatment of SMPP.^[19–22]^

At present, there are a variety of complementary and alternative therapies for SMPP. Randomized controlled trials and systematic reviews have confirmed their respective efficacy, but there is still a lack of effective evidence-based medicine. Therefore, it is necessary to compare a variety of interventions, and then select the best efficacy and safety interventions, to provide effective evidence for the rational selection of clinicians. In this paper, we will conduct a network meta-analysis on the efficacy and safety of various complementary and alternative therapies for SMPP, in order to objectively evaluate the clinical efficacy of different complementary and alternative therapies, and look forward to explore more safe and effective methods to alleviate the symptoms of SMPP and shorten the course of disease.

## 2 Materials and methods

This study will report in strict accordance with PRISMA-P (Preferred Reporting Items for Systematic Review and Meta Analysis Protocols).^[23]^

### 2.1 Study registration

The network meta-analysis has been registered on the International Platform of Registry System Review and Meta-analysis Protocols (INPLASY) and the registration number is INPLASY (https://inplasy.com/inplasy-2020-11-0079/).

### 2.2 Inclusion criteria

#### 2.2.1 Research type

Randomized controlled trials (RCT), whether blind method is used or not, and systematic reviews/meta-analysis of supplementary and alternative therapies for SMPP, including magnetic therapy, acupoint application, cupping, acupuncture, massage, Chinese herbal medicine, etc.

#### 2.2.2 Participants

(1) Children with SMPP are diagnosed.^[24,25]^
(2) Regular use of one or more drugs for the treatment of SMPP.
(3) The age is between 1 and 15 years old.
(4) There are no restrictions on sex and race.

#### 2.2.3 Intervention and comparison

On the basis of conventional western medicine treatment, the treatment group is treated with SMPP supplement and replacement therapy. The basic intervention measures include magnetic therapy, acupoint application, cupping, acupuncture, massage, and Chinese herbal medicine therapy. It can be used alone or in combination. On the basis of conventional western medicine treatment, the control group is treated with other methods except conventional western medicine treatment or intervention measures.

#### 2.2.4 Outcomes

The main outcome measures, the total effective rate = (cured cases + effective cases + markedly effective cases)/total number of cases × 100%.

The curative effect is evaluated according to the following:

(1) Cure. After the treatment, the symptoms of cough and expectoration disappeared, the body temperature is normal, and the body test indexes are normal (lung dry and wet rales and x-ray examination of lung results shadow disappeared).
(2) Significant effect. After the treatment, the symptoms of cough and expectoration are significantly improved, the body temperature is significantly decreased, and the detection indexes of the body are significantly improved (lung dry and wet rales, and shadow of x-ray examination of lung results are reduced).
(3) Effective. After the treatment, the symptoms of cough and expectoration are relieved, the body temperature is basically normal, and the physical indicators gradually returned to normal (lung dry and wet rales, as well as the shadow of x-ray examination of lung results are reduced).
(4) Invalid. After the treatment, the clinical symptoms such as cough and body temperature did not improve or worsen, and other detection indexes did not tend to normal value (lung dry and wet rales, and x-ray examination of lung results shadow did not change or aggravate).

The secondary outcome measures, such as the time to return to normal of clinical symptoms (temperature, cough), length of hospital stay, and incidence of adverse reactions during treatment.

### 2.3 Exclusion criteria

It is necessary to exclude: the next step analysis is not possible due to the design of the research protocol is not rigorous or the data are not complete. There are no clear criteria for efficacy evaluation; repeated published literature. Non clinical controlled trials.

### 2.4 Search strategy

We will search Cochrane Central Register of Controlled Trials, Cochrane Library, PubMed, Web of Science, EMBASE, CNKI, and Wanfang databases to collect RCT studies on complementary and alternative therapies for the treatment of SMPP. At the same time, the relevant references included in the systematic review/meta-analysis are screened, and the retrieval time limit is from the establishment of the database to November 2020. The subject words and free words are used to search. If there are differences in opinion during the process, they can be resolved through relevant discussions or through consultation with a third researcher. The reasons for the differences need to be explained. The specific retrieval strategy of PubMed database is shown in Table 1.

**Table 1:** Detail of the search strategy for PubMed.

No.	Search item
1#	“Pneumonia, Mycoplasma”[Mesh]
2#	((((((((((((((Pneumonia, Primary Atypical[Title/Abstract]) OR (Atypical Pneumonia, Primary[Title/Abstract])) OR (Atypical Pneumonias, Primary[Title/Abstract])) OR (Pneumonias, Primary Atypical[Title/Abstract])) OR (Primary Atypical Pneumonia[Title/Abstract])) OR (Primary Atypical Pneumonias[Title/Abstract])) OR (Mycoplasma Pneumonia[Title/Abstract])) OR (Mycoplasma Pneumonias[Title/Abstract])) OR (Pneumonias, Mycoplasma[Title/Abstract])) OR (Mycoplasma ovipneumoniae Infection[Title/Abstract])) OR (Mycoplasma ovipneumoniae Infections[Title/Abstract])) OR (Mycoplasma pneumoniae Infection[Title/Abstract])) OR (Mycoplasma pneumoniae Infections[Title/Abstract])) OR (Mycoplasma dispar Infection[Title/Abstract])) OR (Mycoplasma dispar Infections[Title/Abstract])
3#	Severe[Title/Abstract]
4#	children[Title/Abstract]
5#	1#OR2#OR3#OR4#
6#	“Complementary Therapies”[Mesh]
7#	((((((((Therapies, Complementary[Title/Abstract]) OR (Therapy, Complementary[Title/Abstract])) OR (Complementary Medicine[Title/Abstract])) OR (Medicine, Complementary[Title/Abstract])) OR (Alternative Medicine[Title/Abstract])) OR (Medicine, Alternative[Title/Abstract])) OR (Alternative Therapies[Title/Abstract])) OR (Therapies, Alternative[Title/Abstract])) OR (Therapy, Alternative[Title/Abstract])
8#	6#OR7#
9#	“Randomized Controlled Trials as Topic”[Mesh]
10#	((Clinical Trials, Randomized[Title/Abstract]) OR (Trials, Randomized Clinical[Title/Abstract])) OR (Controlled Clinical Trials, Randomized[Title/Abstract])
11#	9#OR10#
12#	5#AND8#AND11#

### 2.5 Data extraction

In the process of literature screening, we first screen the title of the article, then read the abstract and the full text, and strictly follow the inclusion criteria and exclusion criteria to determine whether the final inclusion. All extraction process and data are recorded by Microsoft Excel 2019 software.

The specific contents of the final record are as follows: the basic information of the article (including the title of the article, the name of the author, the year of publication, and the publication journal, etc); the characteristics of the subjects (including age, sex, course of disease, diagnostic criteria of SMPP); intervention methods (including intervention measures, basic treatment methods, control measures, and treatment courses); outcome indicators (including main outcome indicators, secondary outcome indicators, and adverse reactions, etc).

### 2.6 Risk of bias assessment

All references to risk bias in the included literature refer to Cochrane Handbook standards.^[26,27]^ It includes random method, allocation concealment, blind method, integrity of data results, selective report of research results, and other factors that may affect authenticity. The judgment of “low” (low bias), “high” (high bias), and “unclear” (lack of relevant information or uncertainty of bias) are made for the above 6 items. The quality of each trial will be independently assessed by 2 researchers.

### 2.7 Statistical analysis

We will use Revman 5.3 software for meta-analysis (https://training.cochrane.org/). The count data are expressed by relative risk ratio (RR) and the measurement data are expressed by mean difference (MD). Both are expressed by 95% confidence interval (CI). Chi-square test is used to analyze the statistical heterogeneity of the included studies (the test level is *α* = 0.1), and the size of heterogeneity is quantitatively judged by combining with *I*^2^. If *P* ≥ .10, *I*^2^ ≤ 50%, there is no heterogeneity, and the fixed effect model is used for analysis; if *P* < .10, *I*^2^ > 50%, it indicates that there is heterogeneity, analyze the causes of heterogeneity, conduct subgroup analysis, and use fixed effect model for analysis; draw funnel diagram to identify whether there is evidence of small sample effect in the included study.

### 2.8 Grading the quality of evidence

We will assess the quality level in terms of risk bias, directness, indirectness, inconsistency, imprecision, and publication bias.^[28]^

## 3 Discussion

MP is an important pathogen of respiratory tract infection in children. Because MP has no cell wall structure, β-lactam and glycopeptide antibiotics acting on cell wall are ineffective. Macrolide antibiotics have been recognized as the first choice of anti-mycoplasma infection drugs at home and abroad because of their long half-life and high drug concentration in the lung.^[29]^ However, with the emergence of Mycoplasma strains resistant to macrolide antibiotics, some children are clinically found to be insensitive to macrolide antibiotics. The clinical symptoms, signs, and imaging findings continue to progress, and SMPP appears. Its treatment is more difficult, and there are even reports of fatal MPP.^[30]^ The selection range of antibiotics for MPP in children is relatively narrow.

Studies have confirmed that complementary and alternative therapies can play a positive role in improving the clinical symptoms of SMPP and shortening the course of the disease. However, traditional meta-analysis lacks a comparison between multiple intervention measures, and may only be limited to the comparison between 2 intervention measures. The purpose of this study is to systematically compare various complementary and alternative therapies for the treatment of SMPP through a network meta-analysis, to further evaluate their respective efficacy and safety.

In this study, although we conducted a comprehensive search of the literature on complementary and alternative treatments for SMPP, there were inevitably some limitations: for example, our study was based on literature reports rather than original data, and some deviations may occur in the process of literature collation. The complementary and alternative therapies of SMPP are easy to operate, non-invasive, economical and easy to be accepted by the families of children. Therefore, through this study, we want to provide help for the treatment of SMPP and provide the basis for rational application of clinicians.

## Author contributions

**Conceptualization:** Xiao Wang, Hongan He, Baoqing Zhang.

**Data curation:** Xiao Wang, Hongan He.

**Formal analysis:** Hongan He, Hao Zheng.

**Investigation:** Hongan He.

**Methodology:** Xiao Wang, Hongan He, Jialin Zheng, Baoqing Zhang.

**Project administration:** Xiao Wang, Hongan He, Baoqing Zhang.

**Search strategy:** Hongan He, Jinjuan Wang, Hao Zheng.**Software:** Hongan He, Jialin Zheng, Jinjuan Wang, Hao Zheng.

**Statistical analysis:** Hongan He, Jialin Zheng.

**Writing – original draft:** Xiao Wang.

**Writing – review & editing:** Baoqing Zhang.
